# Oxidative stress in cervical cancer and its response to chemoradiation

**DOI:** 10.4274/tjod.galenos.2019.19577

**Published:** 2019-07-03

**Authors:** Saiqa Shah, Bhuvanesh Sukhlal Kalal

**Affiliations:** 1 Yenepoya (Deemed to be University), Yenepoya Medical College, Department of Biochemistry, Mangaluru, India; 2 Vydehi Institute of Medical Sciences and Research Centre, Department of Biochemistry, Bengaluru, India; 3 A. J. Institute of Medical Sciences and Research Centre, A. J. Research Centre, Mangaluru, India

**Keywords:** Cervical cancer, copper, malondialdehyde, total antioxidant capacity, chemoradiation

## Abstract

**Objectives:**

Cervical cancer (CaCx) is one of the leading causes of cancer-related death among women worldwide, with the great social and economic burden. Diagnoses in early stages can decrease mortality and morbidity rates. This study was conducted to evaluate the status of serum total antioxidant capacity (TAC), and malondialdehyde (MDA) and copper concentrations among patients with CaCx to determine the level of oxidative stress and effect on which of chemoradiation.

**Materials and Methods:**

Fifty patients with histopathologically proven CaCx who visited the department of oncology & gynaecology and 50 age-matched healthy females were selected for the study. Serum TAC, MDA, and copper were estimated in both study groups. The effect of chemoradiation on these was estimated in patients with CaCx.

**Results:**

The mean ± standard deviation age of the patients was 43.98±6.38 years, whereas that of the controls was 31.56±6.84 years. The mean serum copper and MDA concentrations in the patients was significantly higher as compared with the controls, whereas the mean TAC in the patients was reduced when compared with the controls. After chemoradiation, there was a significant increase and decrease in TAC and MDA, respectively, after chemoradiotherapy, whereas the changes in the copper concentrations were insignificant.

**Conclusion:**

These results suggest that patients with CaCx were in oxidative stress because the oxidative parameters in serum (copper, MDA) were increased and the defensive TAC was decreased in patients with CaCx and chemoradiotherapy improved their anti-oxidant capacity. Further studies are needed to evaluate the concurrent use of antioxidants with chemoradiotherapy for improving the disease prognosis.

**PRECIS:** Oxidative stress among cervical cancer patients found to be increased and chemo-radiotherapy improved their anti-oxidant capacity.

## Introduction

Cervical cancer (CaCx), the third most frequently occurring cancer among women worldwide, accounted for 266,000 deaths in 2012, 87% of which occurred in developing countries^(1)^. CaCx is the leading cause of deaths related to cancer in India and accounts for 17% of all cancer deaths in women aged between 30 and 69 years. Taking current incidence rates into consideration, the annual load of fresh cases is expected to increase to 225,000 by 2025 in India^(2,3)^. The chances of surviving CaCx are considered as 42% in India^(4)^.

The most important causative agent is human papillomavirus (HPV), which spreads through sexual intercourse; males are the carriers in most cases, infecting and generating the disease in women. Many adults are unaware of HPV infection and its associated risks^(5)^.

HPV causes CaCx by damaging DNA, but recent data revealed that oxidative stress plays a role in its development^(6)^. Chemo-radiation is known to improve survival of patients with CaCx. Oxidative stress is a shift of balance towards pro-oxidants in the prooxidant-antioxidant system. Free radicals are generated with a decrease in the levels of antioxidants, which leads to DNA damage, causing dysfunction and disease^(7)^. Severe oxidative stress causes DNA damage and mutations of tumor suppressor genes, initializing events in carcinogenesis, in addition, promoting multi-step carcinogenesis^(7,8,9)^.

Malondialdehyde (MDA) is a known marker of oxidative stress and antioxidant status in patients with cancer. The reactive oxygen species (ROS) produced by different processes initiate lipid peroxidation and lead to the production of excessive levels of MDA, which in turn changes the cellular function and leads to cancer formation^(10)^. The increase in MDA concentration can be attributed to a higher production of ROS due to the increased oxidative damage in patients with uterine cancer. As the disease progresses, the oxygen radical production also increases leading to increased lipid peroxidation. This results in cellular membrane degeneration and DNA damage^(11)^. The increase in free radical generation leads to excessive lipid peroxidation, indicated by a rise in serum MDA. Free radicals may cause evident changes to cell membrane function and the structural organization of DNA, thereby leading to mutations. Therefore, it can be stated that the product of lipid peroxidation could be one of the possible causes of uterine cancer progression^(11,12)^.

The total antioxidant capacity (TAC) measures the antioxidant capacity of whatever antioxidants are present in a biologic sample. It can be used as a dependable biomarker for the diagnosis, prognosis, and prevention of a large number of diseases^(13,14)^.

Copper plays a pivotal role in the oxidant-antioxidant mechanism. The imbalance of copper leads to an increased susceptibility to oxidative damage. Copper acts as a pro-oxidant and may be involved in the formation of free radicals, catalyzed by metal^(15)^. Copper can interact directly with the bases of DNA at the sites of guanine and cytosine^(16)^. *In vitro*, the addition of copper to DNA mediates extensive DNA base damage inducing more mutations^(17)^. Copper also reacts with other free radical species such as hydroxide ion; therefore, the inactivation or loss of certain tumor suppressor genes can lead to the commencement and/or progression of carcinogenesis^(16,17)^. The elevation in copper concentrations may be due to the movement of copper from tissue to serum.

Therefore, the present study evaluated the effect of chemoradiation on serum TAC, and MDA and copper concentrations in patients with CaCx.

## Materials and Methods

The prospective study was conducted between November 2013 and November 2015. Fifty patients with histopathologically proven CaCx who visited the Department of Oncology & Gynaecology, at Vydehi Institute of Medical Sciences and Research Centre were recruited for the study. Subjects with any condition such as severe cardiovascular, respiratory diseases, diabetes mellitus, neurologic and psychiatric disorders, renal disorders and subjects on any medication such as oxidants vitamins, minerals, cigarette smokers, and alcoholics were excluded from the study.

Age-matched healthy women (n=50) visiting the hospital for a routine health check-up and hospital staff members willing to participate in the study were included as healthy controls.

Informed consent was obtained and approximately 3 mL of blood was collected before and after treatment (chemoradiotherapy). Serum was separated from the blood and stored at -40 °C until required for analysis.

Chemotherapy was five cycles of cisplatin weekly in the dose of 40 g/m^2^. Radiotherapy was brachytherapy with four fractions of 7 Gy each and two applications were one week apart.

In both groups, the TAC of serum was estimated using a FRAP assay (ferric reducing antioxidant power or the ferric reducing ability of plasma) according to the method of Benzie & Strain, 1996^(18)^, MDA was measured using the method of Satoh^(19)^, and copper was measured using a modified spectrophotometric micro-method with guanidine hydrochloride^(20)^. In addition, these parameters were estimated in patients with CaCx before and after chemoradiation.

Ethical clearance was obtained from the institutional ethical committee of our institution prior to starting the study (reference no: VIMS & RC/IEC/019/2013-14).

### Statistical Analysis

The results were compiled in an excel spreadsheet, and frequency distribution and Bayesian analysis were performed using the SPSS v 16.0 software package (SPSS, Inc., Chicago, IL, USA).

## Results

In this study, 50 healthy controls and 50 patients with CaCx who fulfilled the eligibility criteria were included in the analysis. The subjects included in the study were females within the age group of 25-65 years. The average age of presentation among the patients was 43.98±6.38 years (Table 1).

**Figure 1:**
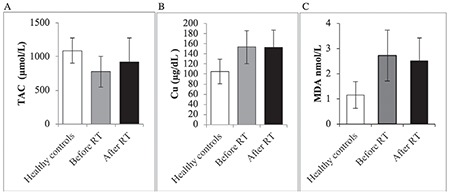
Serum total antioxidant capacity, malondialdehyde, and copper status of health controls and patients with cervical cancer before and after treatment RT: Radiotherapy; data expressed as mean ± SD; **indicates p<0.001; *indicates p<0.05. SD: Standard deviation, TAC: Total antioxidant capacity, MDA: Malondialdehyde, Cu: Copper

The mean serum copper concentration in the CaCx group and control group was 152.96±32.88 µg/dL and 104.88±24.45 µg/dL, respectively. The mean serum TAC in the CaCx group and control group was 781.36±228.88 µmol/L and 1088.94±185.07 µmol/L, respectively. The mean serum MDA concentration in the CaCx group and control group was 2.72±1.01 nmol/mL and 1.17±0.52 nmol/mL, respectively. These results indicated that serum copper and MDA concentrations were significantly (p<0.001) increased in patients with CaCx when compared with the controls, and serum TAC was significantly (p<0.001) decreased in the patient group compared with the controls (Figure 1).

**Figure 2:**
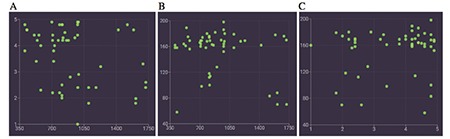
Correlation curve between: TAC-MDA (A), TAC-Cu (B), and MDA-Cu (C) TAC: Total antioxidant capacity, MDA: Malondialdehyde, Cu: Copper

Serum values of TAC, MDA, and copper before andafter chemoradiation were 777.9±227.8 µg/dL, 2.7±1.0 µg/dL, and 153.2±32.6 µg/dL, and 917.8±358.2 µg/dL, 2.5±0.92 µg/dL, and 153±33.7 µg/dL, respectively. Paired sample t-test analysis showed a significant increase and a decrease in TAC (p=0.018), and MDA (p<0.001), respectively, after chemoradiotherapy, whereas the changes in the concentrations of copper, were insignificant (p=0.405). There was a position correction of TAC with copper and MDA with copper (Figure 2).

## Discussion

Exposure of cells to chemotherapy or radiotherapy causes the generation of free radicals and intracellular ROS, which induce cancer cell death. The present study was devised to evaluate and compare the serum values of TAC, MDA, and copper in patients with confirmed CaCx and normal healthy female subjects. There was a negative correlation between serum copper with TAC, and serum TAC with MDA, and a positive correlation between serum copper and MDA.

The role of oxidative stress in the causation of CaCx has been studied extensively. Factors determining the development and progression of CaCx include an imbalance between the detrimental effects of oxidative stress and the antioxidant defense system of the body. In our study, serum copper concentrations were studied in patients with CaCx because copper acts both as pro-oxidant and antioxidant.

Copper in its free, unbound form catalyzes the production of various toxic free radicals^(15)^. Elevated copper concentrations have the potential to produce a relatively continuous supply of free radicals and ROS formed within cells are highly reactive and are able to oxidize most of the biomolecules within the cell, leading to tissue injury and cancer. ROS have been associated for many years with oncogenesis; however, recently a new role has emerged for ROS as mediators of signaling pathways, leading to cell proliferation and tumor initiation and promotion^(21)^.

In our study, we obtained a significant (p<0.001) increase in serum copper concentrations in patients as compared with controls. Our results of high serum copper in patients with CaCx are also in agreement with several studies that found increased concentrations of serum copper in subjects with CaCx when compared with healthy controls^(6,22,23,24)^. Although, the cause of the increase in serum copper concentrations among patients with cancer is not known, it was proposed to be related with the increased liver production of copper-containing ceruloplasmin as an inflammatory response to cancer or from a tumor-induced decrease in the catabolism of the serum ceruloplasmin^(22)^. The meta-analysis by Zhang et al.,^(23)^ showed significant evidence of higher serum copper concentrations in patients with CaCx than in controls, suggesting that serum copper exposure was a risk factor in CaCx. The increased copper level could be related to the fact that copper is needed to form new blood vessels and because cancer needs them in order to grow^(25)^. Hence, the increase in copper values in patients with CaCx.

The other finding of our study was a significant (p<0.001) decrease in serum TAC in patients as compared with controls. These results were in line with those of Demirci et al.,^(26)^ and Rong et al.,^(27)^ who reported the altered antioxidant status of patients with CaCx compared with healthy controls. However, Lee et al.,^(28)^ stated that cervical intraepithelial neoplasia was also associated with lower blood antioxidant capacity levels. Kim et al.,^(29)^ found that CIN was also associated with lower blood antioxidant capacity levels. The human body contains a complex antioxidant defense system that depends on the dietary intake of antioxidants as well as the endogenous production of anti-oxidative compounds such as glutathione^(30)^.

Antioxidants are cytoprotective chemicals that prevent oxidative damage caused by free radicals. ROS and nitrogen species, which are oxygen and nitrogen-derived free radicals, are generated naturally as by-products of cellular metabolism^(15)^. Under physiologic conditions, free radicals are immediately rendered inactive by antioxidants. However, in oxidative stress, these free radicals remain in excess and cause damage by reacting with macromolecules such as nucleic acids, proteins, polyunsaturated fatty acids,  and carbohydrates^(30)^. Oxidative damage has been associated with the pathogenesis of many chronic medical disorders viz. atherosclerosis, cancer, arthritis, and neurodegenerative disorders^(31)^. DNA damage and abnormal DNA repairs are prime factors in the causation of cancer. Hence, the association between a low antioxidant level and various malignant and premalignant conditions has been assessed by researchers.

Further, we showed a significant increase (p<0.001) in serum MDA concentrations in patients with CaCx. Grace et al.,^(32)^ estimated MDA in patients with CaCx to assess the extent of lipid peroxidation and found similar results of increased circulating levels of MDA. They attributed it to an increase in oxidative stress due to a deficiency of antioxidant mechanism. They also observed an increase in lipid peroxidation and a decline in enzymatic antioxidant status in patients with CaCx. Naidu et al.,^(6)^ also found increased levels of MDA and suggested that it was a possible cause in the progression of CaCx. The same results of elevated MDA concentrations were found by Demirci et al.,^(26)^ in patients with CaCx when compared with a control group. As per Demirici et al.,^(26)^ oxidative damage leads to the formation of products such as MDA, and DNA damage, which may, in turn, lead to mutagenesis, carcinogenesis, and cell death.

### Study Limitations

There are some limitations in this study. This is a single centre hospital based study with small number of patients, the assessment of other oxidative enzymes, nutritional status are needed with larger patients size to get the solid evidence of antioxidants levels after receiveing the chemoradiotherapy.

## Conclusion

The present findings demonstrate the imbalance in serum TCA, MDA, and copper among patients with CaCx when compared with healthy controls. These changes may play an important role in the pathogenesis and progression of CaCx through the involvement of these parameters in exaggerated oxidative stress. Further, chemoradiotherapy improved its anti-oxidant capacity. Extended studies are needed to evaluate the concurrent use of antioxidants with chemoradiotherapy for improving the disease prognosis.

## Figures and Tables

**Table 1:**
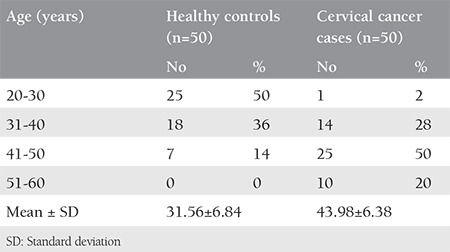
Age distribution of healthy controls and cervical cancer (CaCx) patients
